# A streamlined, resource-efficient immunoprecipitation-mass spectrometry method for quantifying plasma amyloid-β biomarkers in Alzheimer’s disease

**DOI:** 10.21203/rs.3.rs-4947448/v1

**Published:** 2024-09-02

**Authors:** Thomas Karikari, Yijun Chen, Xuemei Zeng, Marcos Olvera-Rojas, Anuradha Sehrawat, Tara Lafferty, Tharick Pascoal, Victor Villemagne, Patricio Solis-Urra, Eva Triviño-Ibañez, Manuel Gómez-Rí, Ann Cohen, Milos Ikonomovic, Irene Esteban-Cornejo, Kirk Erickson, Oscar Lopez, Nathan Yates

**Affiliations:** University of Pittsburgh; University of Pittsburgh; University of Pittsburgh; University of Granada; UPMC Children’s Hospital Of Pittsburgh; University of Pittsburgh; University of Pittsburgh; University of Pittsburgh; University of Granada; Hospital Universitario Virgen de las Nieves; Hospital Universitario Virgen de las Nieves; University of Pittsburgh; University of Pittsburgh; University of Granada; University of Pittsburgh; University of Pittsburgh; University of Pittsburgh

**Keywords:** Alzheimer’s disease, blood biomarkers, amyloid beta, immunoprecipitation, mass spectrometry

## Abstract

High-performance, resource-efficient methods for plasma amyloid-β (Aβ) quantification in Alzheimer’s disease are lacking; existing mass spectrometry-based assays are resource- and time-intensive. We developed a streamlined mass spectrometry method with a single immunoprecipitation step, an optimized buffer system, and ≤75% less antibody requirement. Analytical and clinical performances were compared with an in-house reproduced version of a well-known two-step assay. The streamlined assay showed high dilution linearity (r^2^>0.99) and precision (< 10% coefficient of variation), low quantification limits (Aβ1–40: 12.5 pg/ml; Aβ1–42: 3.125 pg/ml), and high signal correlation (r^2^~0.7) with the two-step immunoprecipitation assay. The novel single-step assay showed more efficient recovery of Aβ peptides via fewer immunoprecipitation steps, with significantly higher signal-to-noise ratios, even at plasma sample volumes down to 50 pl. Both assays had equivalent performances in distinguishing non-elevated vs. elevated brain Aβ-PET individuals. The new method enables simplified yet robust evaluation of plasma Aβ biomarkers in Alzheimer’s disease.

## Introduction

Brain amyloid β (Aβ) deposition is a pathological hallmark and diagnostic criterion of Alzheimer’s disease (AD) [1, 2]. Following the recent approval of anti-Aβ monoclonal antibody therapies by the United States Food and Drugs Administration (FDA), the importance of reliable yet accessible biomarkers in clinical settings has become increasingly essential [3–5]. Currently, the most widely used biomarkers for assessing Aβ deposition are positron emission tomography (PET) imaging of Aβ plaques, and cerebrospinal fluid (CSF) measurements of Aβ42/40 peptide ratio via immunoassays [6, 7]. However, these methods are limited by their high costs, invasiveness, and lack of widespread availability, which restrict their use in routine clinical assessments [8]. Blood-based biomarkers, such as the plasma Aβ1–42/1–40 ratio, offers a minimally invasive and affordable option that may guide, complement, or serve as a substitute for PET and CSF testing [9–11].

Several efforts have focused on developing assays for plasma Aβ biomarkers [1, 12–16]. These assays mostly employ either immunoassay [17–20] or immunoprecipitation-mass spectrometry (IP-MS) [21–25] methods. Although immunoassays are used for Aβ measurement, their susceptibility to non-specific interference can compromise accuracy, particularly at the low plasma Aβ peptide levels typically found in the early AD stages [20, 26]. Conversely, sensitive IP-MS assays have demonstrated higher effectiveness in distinguishing individuals with and without brain Aβ plaque pathology, evidenced by decreased plasma Aβ1–42/1–40 ratio [27, 28] but tend to be more expensive and less scalable.

Several groups have reported IP-MS assays that detect Aβ peptides in plasma samples [21–25]. In 2014, Pannee et al. initially quantified plasma Aβ1–42, Aβ1–40, and Aβ1–38 in a small cohort [29], and later in a larger cohort [24]. In the same year, Kaneko et al. demonstrated high sensitivity and specificity of the APP669–711/Aβ1–42 ratio for brain amyloidosis as measured by Aβ PET [30]. In 2017, Ovod et al. introduced a novel plasma Aβ assay, finding significantly lower Aβ1–42 concentrations and Aβ1–42/Aβ1–40 ratios in Aβ PET-positive patients compared with Aβ PET-negative participants [28]; these findings were extended in a later study by Schindler et al [21].

In 2018, Nakamura et al. presented a matrix-assisted laser desorption/ionization (MALDI) time-of-flight (TOF) MS method to measure Aβ peptides from 250 μl of plasma. This assay demonstrated robust predictive capabilities of both the Aβ1–40/Aβ1–42 and APP669–711/Aβ1–42 ratios for assessing brain Aβ burden [22]. Subsequent studies revealed potential clinical utility of the assay in predicting individual brain Aβ burden and discriminating AD from non-AD neurodegenerative conditions [31].

Recognizing the importance of plasma Aβ measurement for AD detection, we previously adapted the IP-MS assay originally developed by Nakamura et al., referred to here as the Pittsburgh plasma Aβ assay version 1.0 (PAβ V1.0) [32]. The PAβ V1.0 assay was implemented on a cost-effective benchtop MALDI-TOF instrument, the Bruker Microflex LT. However, considering the limited dynamic range of MS, the presence of strong background interference including albumin and immunoglobulins could impede the accurate detection of plasma Aβ peptides [14, 33]. To address this limitation, the V1.0 assay uses two rounds of immunoprecipitation (IP). Regrettably, this approach increases per-sample costs, reagent usage, and sample preparation time.

Here, we describe a novel IP-MS assay, referred to as the Pittsburgh Plasma Aβ assay version 2.0 (PAβ V2.0). This new version streamlines the two steps of IP into one, using a commercially available supplement buffer, which was identified in a screen of buffers and detergents that can effectively improve the signal-to-noise ratio (S/N). We evaluate the analytical and clinical performance compared with the PAβ V1.0 assay, focusing on the biomarkers Aβ1–42, Aβ1–40, and the Aβi–42/Aβi–40 ratio.

## Material and Methods

### 2.1 Participants recruitment

This study included plasma samples from two cohorts. For the first cohort, we enrolled participants from the University of Pittsburgh Alzheimer’s Disease Research Center (ADRC) in Pittsburgh, Pennsylvania, USA. The participants in this ongoing study undergo annual clinical evaluation to assess their longitudinal brain health and potential development of cognitive impairment and dementia. Annual evaluations include neuroimaging, cognitive testing, and blood collection for use in plasma biomarker analysis outside of the clinical assessment. Neuropsychological evaluation and diagnoses were established through clinical assessments [28, 29]. The battery of cognitive tests included the Montreal Cognitive Assessment (MoCA), Mini-Mental State Examination (MMSE), and the Clinical Dementia Rating (CDR) scale. The current investigation was a prospective, blinded sub-study where participants were enrolled based on their order of clinical attendance and their informed consent to participate. This involved agreeing to provide an additional tube of blood for the project. The ADRC study was approved by the University of Pittsburgh Institutional Review Board (MOD19110245–023).

The second cohort was sourced from the Active Gains in Brain Using Exercise During Aging (AGUEDA) project (NCT05186090). Participants were recruited from Granada, Spain, based on their classification as physically inactive and cognitively normal, assessed by the Spanish Telephone Interview for Cognitive Status modified (STICS-M), MMSE, and MoCA. As an outcome, Aβ PET was performed using the [^18^F] Florbetaben tracer, quantified using standardized uptake value ratio (SUVR) values and the Centiloid (CL) scale. Detailed information on eligibility criteria, participant selection methods, and recruitment procedures, as well as details about the study setting, locations, and data collection, can be found in a comprehensive description provided in the AGUEDA protocol [34]. Prior to enrollment in the AGUEDA trial, participants provided informed consent, and the trial was conducted in accordance with the approval of the Research Ethics Board of the Andalusian Health Service (CEIM/CEI Provincial de Granada; #2317-N-19). In this cross-sectional analysis, we focused on the baseline data.

Researchers were blinded to all participant information until the completion of data acquisition.

### 2.2 Blood collection and processing procedures

At the University of Pittsburgh ADRC, blood samples were collected via venipuncture by nurses with extensive clinical experience and trained in ADRC procedures [35]. Blood collection was performed between 9:00 am and 2:00 pm, with the time of last meal recorded. For the AGUEDA cohort, blood samples were collected at 08:00–10:00 am following longer than 8 hours of fasting, at the Virgen de las Nieves University Hospital, Spain.

Briefly, a 10 and 4 ml Lavender top ethylenediaminetetraacetic acid (EDTA) tube was used to collect whole blood from each participant in the ADRC and AGUEDA cohort, respectively. Following each blood draw, the tubes were promptly inverted 8 to 10 times and subsequently centrifuged at 2000 xg for 10 minutes for the AGUEDA cohort and 15 minutes for the ADRC cohort at 4°C to effectively separate the plasma. The resulting plasma samples were aliquoted into cryovials and frozen at −80°C until use, following standard guidelines [35].

### 2.3 Immunoaffinity enrichment

#### Pittsburgh plasma Aβ assay (PAβ) V1.0

The PAβ V1.0 assay was developed at the University of Pittsburgh based on the method originally described by Nakamura et al [22]. For each sample, 250 μl of binding buffer (100 mM Tris-HCl pH 7.4 [Sigma #T2788–1L], 300 mM NaCl [Sigma #S7653–250G], 0.2% w/v n-dodecyl-β-D-maltoside [DDM; Sigma #D4641–1G], 0.2% w/v n-nonyl-β-D-thiomaltoside [NTM; Anatrace #148565–55-3]) containing 62.4 pg/ml of Aβ1–38 internal standard (IS) (Anaspec #AS-65220), was added to a 1.5 ml Eppendorf Protein LoBind Tube (ThermoFisher #13–698-794), followed by the addition of 250 μl plasma sample. To facilitate direct comparison with the PAβ V2.0 assay, 100 pg/ml Aβ1–40 IS (Rpeptide #A-1101–2) and 30 pg/ml Aβ1–42 IS (Rpeptide #A-1102–1) were also added to the binding buffer for the evaluation of analytical performance.

**Figure 1:**
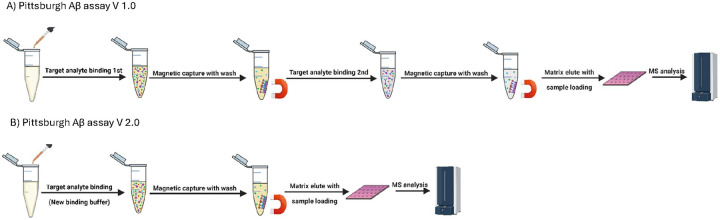
Schematic illustration of the PAβ V1.0 vs PAβ V2.0 assays. The PAβ V1.0 assay protocol (A) entails two rounds of immunoprecipitation. In contrast, the PAβ V2.0 assay protocol (B) includes a simplified sample preparation procedure with only a single round of IP, saving time and resources.

The samples were immunoprecipitated with 10 μl of 50 mg/ml Dynabeads (M-270 Epoxy; ThermoFisher #14301) coupled with 5 μg 6E10 anti-Aβ antibody (BioLegend #803003) for 1 hour at 4°C with rotation. The beads were coupled with the antibody following the protocol recommended by the manufacturer. After the IP, the supernatant was discarded, and the beads washed once with 0.5 ml of cold phosphate-buffered saline (PBS, Gibco #2537136). The washed beads were then transferred to a fresh Eppendorf tube using 0.5 ml of cold PBS and eluted with 25 μl of glycine elution buffer (50 mM glycine [pH 2.8, Sigma #G2879–100G], 0.1% DDM) after removing all liquid. The eluates were collected and transferred to fresh tubes containing 0.5 ml of the binding buffer (without any Aβ ISs) for a second round of IP. Following one hour of rotation at 4°C, the beads were washed twice with 0.5 ml of cold HPLC-grade H_2_O (Fisher #7732–18-5) and transferred to a fresh Eppendorf tube by resuspending in 0.2 ml H_2_O. After complete removal of all liquid through vacuum aspiration, the beads were eluted using 6 μl of 3 mg/ml α-cyano-4-hydroxycinnamic acid matrix (Bruker #8201344) dissolved in TA50 (50% Acetonitrile [Fisher #75–05-8], 0.1% Trifluoroacetic acid [Alfa Aesar #UN2699], 1 mM ammonium dihydrogen phosphate [Sigma #204005]). The eluate was spotted four times with 1 μl each onto the MALDI target plate (Bruker #8280823) for MS analysis. A schematic illustration of the workflow for this assay is shown in Fig. 1A.

### Single IP procedure for detergents and blocking buffer tests

Similar to the first IP step of the PAβ V1.0 assay, we prepared 250 μl of the same assay binding buffer, either used as is or supplemented with one of the following detergents or blocking buffers: 10% v/v SuperBlock (Thermo #37535), 10 μg/ml TruBlock (Meridian #A66803H), 0.5% v/v Triton100 (Millipore #648462), 0.5% v/v Tween20 (BioRad #1610781), or 10% Quanterix Neurology Plex 4E CSF sample diluent (N4PE CSF diluent [Quanterix #103727]) for different tests.

This mixture was transferred to a 1.5 ml Eppendorf Protein LoBind tube with 62.4 pg/ml of Aβ1–38 IS, 100 pg/ml of Aβ1–40 IS, and 30 pg/ml of Aβ1–42. Subsequently, 250 μl of human plasma sample was added to the mixture. The sample was immunoprecipitated with 5 μl of 50 mg/ml Dynabeads coupled with 1.25 μg 6E10 Aβ antibody (BioLegend #803003) for 1 hour at 4°C with rotation. After IP, the supernatant was discarded, and the beads resuspended in 0.5 ml of the assay binding buffer with the corresponding supplement added as appropriate and transferred to a new tube. The beads underwent an additional wash with 0.5 ml of the binding buffer with corresponding supplement, two washes with 0.5 ml of PBS and one wash with 0.5 ml of HPLC-grade H_2_O. Finally, the beads were transferred to a fresh Eppendorf tube using 0.2 ml of H_2_O. After removal of all liquid through vacuum aspiration, the beads were eluted using 6 μl of 3 mg/ml α-cyano-4-hydroxycinnamic acid matrix dissolved in TA50. The eluate was spotted four times with 1 μl each onto the MALDI target plate for analysis.

#### Screening of buffers and blockers for the PAβ V2.0 assay

We evaluated the effects of several buffer systems and heterophilic blocking agents for the PAβ V2.0. These included the 10% N4PE CSF diluent from Quanterix, the 10% v/v SuperBlock, 10 μg/ml TruBlock, 0.5% v/v Triton100 and 0.5% v/v Tween20. The results from the PAβ V2.0 assay were compared to those obtained using the PAβ V1.0 assay.

### 2.4 MALDI-TOF MS

After sample spotting, the MALDI target plate was air dried and then loaded into a benchtop MALDI- TOF mass spectrometer, Microflex LT (Bruker Daltonics), equipped with a 337 nm nitrogen laser to acquire mass spectra. The Microflex LT operated in linear mode with a pulsed positive ion extraction setting, utilizing an attenuator offset of 12%, an attenuator range of 30%, and 63% laser power. An external mass calibration was performed using a peptide calibration mixture consisting of two calibration standards (Bruker #8222570, #8206355). The auto scan function was utilized, acquiring one spectrum for each spot through the combination of ion signals from 2,500 laser shots, resulting in four spectra per sample. Aβ1–38 IS was employed to ensure spectrum quality in the auto scan function. Only spectrum, generated from every 50 shots, with Aβ1–38 IS S/N ratios greater than three were collected. After acquisition, the spectra underwent smoothing using the SavitzkyGolay algorithm with a width of 0.1 mass-to-charge (m/z) and baseline subtraction using the TopHat algorithm. The peak intensity and S/N ratios were measured using FlexControl (v3.4, Bruker Daltonics). Subsequently, ClinPro Tools Software (v2.1, Bruker Daltonics) was employed for m/z alignment, peak detection, and peak area calculation.

### 2.5 Analytical assessment

Linearity analysis was conducted using a two-fold serial dilution of an Aβ peptide mixture, starting with concentrations of 400 pg/ml for Aβ1–40 (Anaspec, #AS-24235) and 10 pg/ml for Aβ1–42 (Anaspec, #AS-20276), in 6% bovine serum albumin (BSA)/PBS, diluting up to 64x. The analysis involved six replicates for each dilution, totaling 36 samples, which were evenly processed across two batches. The lower limit of quantification (LLOQ) was established as the lowest concentration measurable with a coefficient of variation (CV) under 20% [25]. The working range was defined as the range from the LLOQ to the highest concentration tested. To evaluate the plasma matrix effect, we assessed the recovery by comparing the results in plasma to those in 6% BSA/PBS at three different concentration levels. Both media were spiked with equal amounts of Aβ1–40 and Aβ1–42 prior to the IP procedures. Recovery was calculated using the formula:

\%Recovery=100\%×(P_spiked plasma\-−P_plasma)/P_spiked BSA

where P represents the normalized peak area. Intra- and inter-assay variability were determined by analyzing samples at three Aβ concentrations levels across five batches, each containing six replicates per concentration.

The linearity, LLOQ, working range, matrix effect recovery and precision of Aβ1–40 and Aβ1–42 were normalized using either common IS (Aβ1–38 IS) or analyte specific IS (Aβ1–40 IS and Aβ1–42 IS), respectively.

### 2.6 Plasma dilution linearity

The effect of plasma dilution on normalized intensity for both the PAβ Vi.0 and PAβ V2.0 assay formats were investigated by testing five separate amounts of a pooled plasma sample (50 μl to 250 μl), with three replicates each. All samples in this test were diluted to 250 μl prior to processing, and Aβ1–40 and Aβ1–42 levels were normalized using the Aβ1–38 IS only.

### 2.6 Simoa assay for IP recovery assessment

To quantify the proportion of Aβ peptides retained after the IP procedures, Single Molecule Array (Simoa) assays were utilized. These assays were performed using the Simoa Human Neurology 4-Plex E assay (N4PE) kit from Quanterix (#i03670) on an HD-X analyzer (Quanterix, Billerica, MA, USA). To monitor assay performance, quality control samples at three different concentrations were analyzed at the beginning and end of each assay run. The average %CV for the quality controls was below 5%.

Mass spectrometric and immunoassay experiments were performed in the Mass Spectrometry facility at the Biofluid Biomarker Laboratory, Department of Psychiatry, School of Medicine, University of Pittsburgh, Pittsburgh, PA, USA.

### 2.7 Clinical Performance Assessment

We compared three different Aβ biomarkers: Aβ1–42/Aβ1–40 using the PAβ Vi.0 assay, Aβ1–42/Aβ1–40 using the PAβ V2.0 assay, and Aβ1–42/Aβ1–40 normalized with the Aβ1–42 IS and the Aβ1–40 IS correspondingly using the PAβ V2.0 assay. The evaluation of biomarker performance was conducted across the PITT-ADRC based on the clinical assessments for cognitive status (ADRC cohort), and the AGUEDA cohort based on the Aβ PET imaging results (AGUEDA cohort) using CL scales (AGUEDA cohort).

The assay performance over multiple batches was evaluated using pooled quality control plasma samples at two concentration levels by measuring the Aβ1–40 and Aβ1–42. In both assays, normalization of Aβ1–40 and Aβ1–42 was conducted using the Aβ1–38 IS. The intra- and inter-assay %CV were determined to be less than i5% for both cohorts.

### 2.8 Correlation Analysis

The correlation between the PAβ Vi.0 and PAβ V2.0 assay formats was evaluated using the normalized peak areas of multiple Aβ biomarkers, including Aβ1–42, Aβ1–40, Aβ1–39, Aβ3–40, Aβ1–38, and APP669–7ii, across the PITT-ADRC and AGUEDA cohorts. All analytes were normalized using Aβ1–38 as the IS. Additionally, Aβ1–42 and Aβ1–40 signals in the PAβ V2.0 assay format were further normalized using their respective IS; Aβ1–42 IS and Aβ1–40 IS.

### 2.9 Statistical Analysis

For participant demographic characteristics, continuous variables were summarized using means and standard deviations, while categorical variables were reported as numbers and percentages. Differences across cohorts for continuous variables were examined using the Wilcoxon Rank Sum test or Kruskal-Wallis test, depending on the number of groups involved. Categorical variables were analyzed using Fisher’s exact tests. For S/N ratio comparison between different assays, Wilcoxon Rank Sum test was used. For clinical assessments, box and whisker plots were generated using clinical assessments, Aβ PET imaging results, and CL scales over the cohorts. Wilcoxon Rank Sum test was used to assess the disease discriminating performance of biomarkers across cohorts based on the clinical assessments or the Aβ PET imaging results. The Kruskal-Wallis test was used to evaluate the difference among the CL scale groups. The Cohen’s d was calculated for multiple assay biomarkers to evaluate the standardized difference between different diagnostic groups. For correlation study, Spearman correlation analysis was conducted to evaluate the strength of the association between Aβ peptide measurements from the two different assays. For all the tests, a p-value less than 0.05 was considered statistically significant. All analyses were performed using R statistical software (version 4.2.1, R Foundation for Statistical Computing, Vienna, Austria), available at [http://www.r-project.org/].

## Results

### 3.1 Effectiveness of detergents and blocking buffers in reducing IP-MS background

To streamline the PAβ V1.0 assay into a single IP step, we experimented with various supplements in the IP binding buffer to reduce background interference. These included 10% N4PE CSF diluent, 10% SuperBlock, 10 μg/ml TruBlock, 0.5% Triton100, and 0.5% Tween20, were all tested following the *Single IP protocol* (see Methods section).

**Figure 2:**
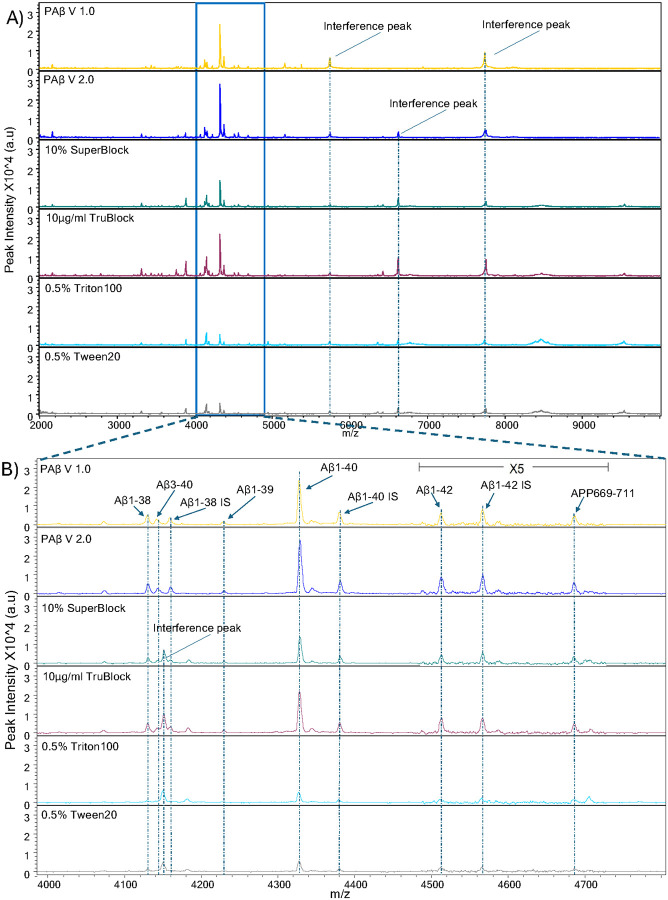
Mass spectra representative of Aβ peptides over multiple reagents and blocking buffers. (A) MALDI-TOF mass spectra of plasma Aβ peptides replicates utilizing the PAβ V1.0 assay procedure, comparing five different buffers or detergents using single IP procedure; 10% N4PE CSF diluent (PAβ V2.0 assay), 10% SuperBlock, 10μg/ml TruBlock, 0.5% Triton100, and 0.5% Tween20. Representative spectra from each experiment are presented. Interference peaks were consistently observed at 5771.1 m/z and 7746.8 m/z across all assays. Additionally, another interference peak at 6631.0 m/z was consistently noted in all assays except the PAβ V1.0 assay. (B) Upon magnification in the range of 4000–4850 m/z, the theoretical m/z values of peptides were as follows: 4132.6 m/z for Aβ1–38, 4144.7 m/z for Aβ3–40, 4231.8 m/z for Aβ1–39, 4330.9 m/z for Aβ1–40, 4515.1 m/z for Aβ1–42, and 4689.4 m/z for APP669–711. Aβ1–38 IS at 4160.7 m/z, Aβ1–40 IS at 4383.3 m/z, and Aβ1–42 IS at 4569.3 m/z were utilized as internal standards for the normalization of mass spectra. Notably, an interference peak was detected at 4153.4 m/z in samples processed using 10% SuperBlock, 10pg/ml TruBlock, 0.5% Triton100, and 0.5% Tween20.

Among the supplements tested, the N4PE CSF diluent demonstrated the best performance, effectively eliminating interference peaks while maintaining the highest S/N ratio. Consistent with the PAβ V1.0 assay, the interference peak at 4450 m/z, which often obscures the Aβ1–38 and Aβ3–40 signals, was significantly eliminated with the use of the N4PE CSF diluent but not with the other supplements. Notably, SuperBlock and TruBlock resulted in significantly lower S/N ratios when compared with the PAβ V1.0 assay. The detergents, on the other hand, showed the lowest S/N ratios for all Aβ peptides. (Fig. 2 and Supplementary Fig. 1).

**Figure 3:**
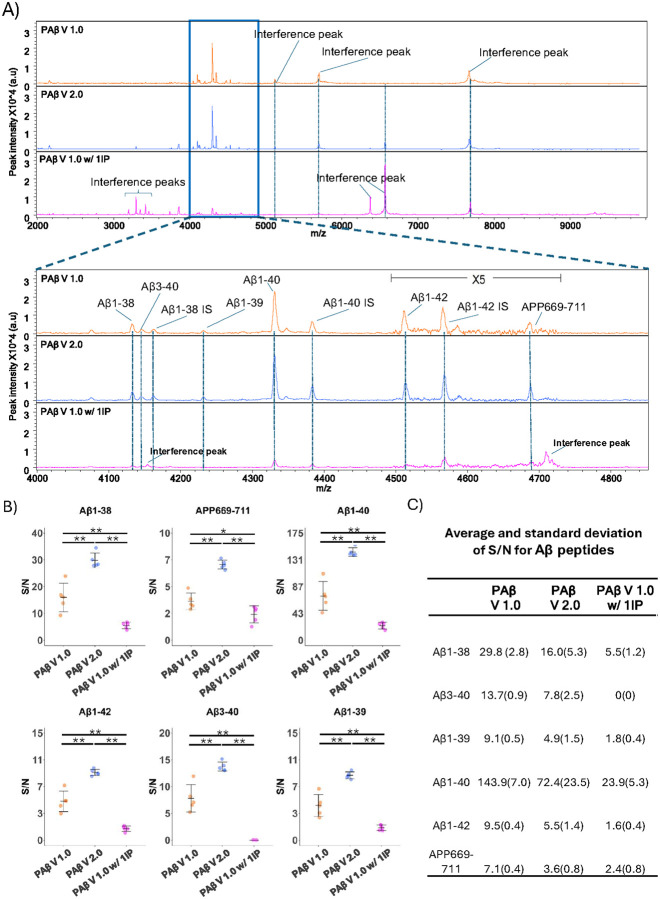
The spectra of IP-MS assays with S/N comparison. (A) MALDI–TOF mass spectra of Aβ peptides derived from plasma replicates utilizing the PAβ V1.0 assay, 10% N4PE CSF diluent (PAβ V2.0 assay) and PAβ V1.0 assay with 1IP. Representative spectra from each experiment are presented. Interference peaks were consistently observed at 5771.1 m/z and 7746.8 m/z across all assays. Additionally, another interference peak at 6631.0 m/z was consistently noted in all assay formats except the PAβ V1.0 assay. Interference peaks at 3200 m/z to 3500 m/z and 6432.4 m/z were observed in PAβ Vi.0 assay with 1IP only. Upon magnification to the range of 4000–4850 m/z, the theoretical m/z values of peptides are as follows: 4i32.6 m/z for Aβ1–38, 4i44.7 m/z for Aβ3–40, 4231.8 m/z for Aβ1–39, 4330.9 m/z for Aβ1–40, 45i5.i m/z for Aβ1–42, and 4689.4 m/z for APP669–711. Aβ1–38 IS at 4160.7 m/z, Aβ1–40 IS at 4383.3 m/z, and Aβ1–42 IS at 4569.3 m/z were utilized as internal standards for the normalization of mass spectra. Notably, an interference peak was detected at 4153.4 m/z in samples processed using PAβ V1.0 assay with 1IP but not in the other assays. (B) S/N ratios were compared across three assays in triplicates, with asterisks indicating significant differences (*p < 0.05, **p < 0.0i) as determined by the Wilcoxon Rank Sum test. (C) The averages and standard deviations of the S/N ratios are listed.

As a confirmation comparison, the original PAβ V1.0 assay and the single IP assay that used the N4PE CSF diluent were compared with the original PAβ V1.0 assay configuration with one IP step and no binding buffer supplementation. As shown in the representative spectra (Fig. 3A), supplementing the IP binding buffer with the N4PE CSF diluent resulted in the cleanest spectra. Similar to the PAβ V1.0 assay, the interference peaks observed in the PAβ V1.0 assay with a single round of IP at 3200 m/z to 3500 m/z, and at 6400 m/z and 6600 m/z, were reduced by using the N4PE CSF diluent. Furthermore, the single round of IP procedure using N4PE CSF diluent achieved a significantly higher S/N ratio, with means of 143.9 for Aβ1–40 and 9.5 for Aβ1–42, compared with 72.4 and 5.5 respectively in the PAβ V1.0 assay, and 23.9 and 1.6 in the PAβ V1.0 assay with one IP (Figs. 3B, 3C). Similar improvements were also observed for other Aβ peptides. In the PAB V1.0 assay, the S/N ratios were 16.0, 7.8, 4.9, and 3.6 in PAβ V1.0 assay and 5.5, 0, 1.8, and 2.4 in the PAβ V1.0 assay with a single IP for Aβ1–38, Aβ3–40, Aβ1–39, and APP669–711, respectively. Conversely, in the single IP with N4PE CSF diluent, these ratios improved to 29.8, 13.7, 9.1, and 7.1 for the same peptides (Figs. 3B, 3C).

Due to the optimal performance, we selected the single IP with N4PE CSF diluent-supplemented binding buffer as the Pittsburgh assay PAβ V2.0.

### 3.2 Analytical assessment

We proceeded to compare the analytical performance of the PAβ V1.0 assay with the PAβ V2.0 assay.

#### Linearity test, LLOQ, ULOQ and Assay range

**Figure 4:**
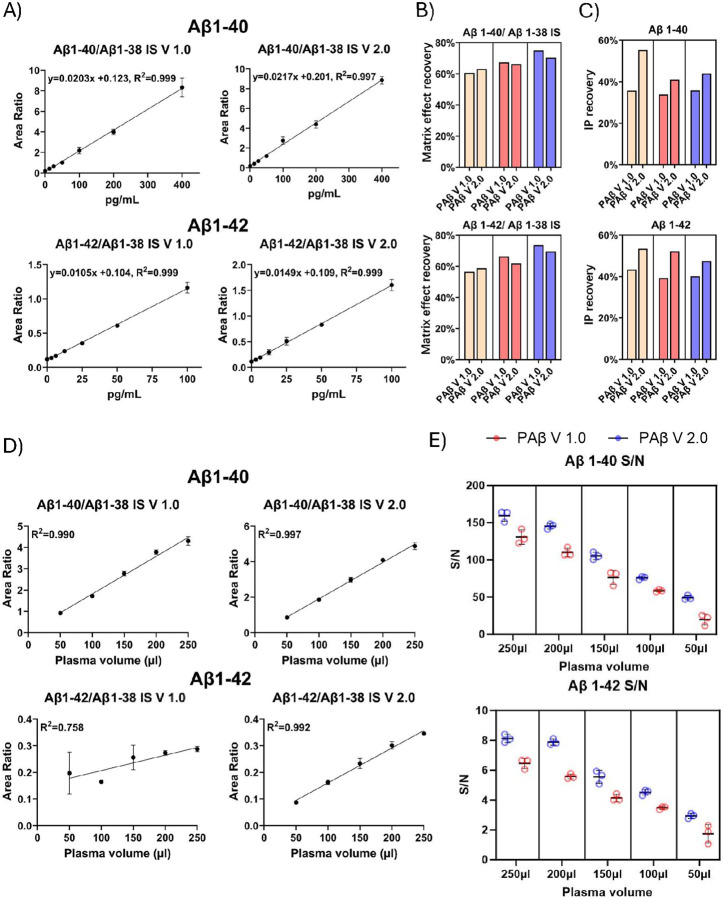
Analytical performance assessment of the IP-MS assays. (A) The calibration curves were generated using Aβ1–40 concentrations of 400pg/ml, 200pg/ml, 100pg/ml, 50pg/ml, 25pg/ml, 12.5pg/ml, and 0pg/ml, and Aβ1–42 concentrations of 100pg/ml, 50pg/ml, 25pg/ml, 12.5pg/ml, 6.25pg/ml, 3.125pg/ml, and 0pg/ml, normalized with Aβ1–38 IS. (B) The matrix effect recovery was assessed across three different concentrations, each with three replicates, utilizing Aβ1–38 IS normalization. (C) The IP recovery was measured through the SIMOA assay. (D) The relationship between plasma dilution and normalized intensity of the PAβ V1.0 and PAβ V2.0 assays. Three replicates were performed for each volume. Both Aβ1–40 and Aβ1–42 were normalized by Aβ1–38 IS. (E) The S/N ratios of plasma samples with various volumes were compared between PAβ V1.0 and PAβ V2.0 assays for Aβ1–40 and Aβ1–42.

**Figure 5:**
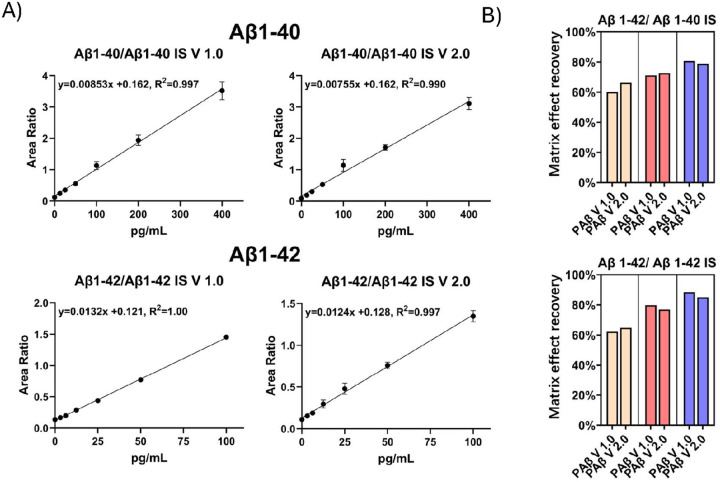
Calibration curve and matrix effect recovery assessment using Aβ1–40 IS and Aβ1–42 IS normalization. (A) The calibration curves were generated using Aβ1–40 concentrations of 400pg/ml, 200pg/ml, 100pg/ml, 50pg/ml, 25pg/ml, 12.5pg/ml, and 0pg/ml, and Aβ1–42 concentrations of 100pg/ml, 50pg/ml, 25pg/ml, 12.5pg/ml, 6.25pg/ml, 3.125pg/ml, and 0pg/ml, normalized with Aβ1–40 IS and Aβ1–42 IS. (B) The matrix effect recovery was assessed across three different concentrations, each with three replicates, utilizing Aβ1–40 IS and Aβ1–42 IS normalization.

To assess linearity, we constructed standard curves using two-fold serial dilutions of a mixture of Aβ1–40 and Aβ1–42 in 6% BSA/PBS. A total of seven samples containing varying concentrations of Aβ1–40 (0.00 pg/ml, 12.5 pg/ml, 25.0 pg/ml, 50pg/ml 100 pg/ml, 200 pg/ml, 400 pg/ml) and Aβ1–42 (0.00 pg/ml, 3.125 pg/ml, 6.25 pg/ml, 12.5 pg/ml, 25.0 pg/ml, 50 pg/ml and 100 pg/ml) were included in the linearity test. The measured Aβ1–40 and Aβ1–42 peak areas were normalized using the Aβ1–38 IS (Fig. 4A) or the analyte specific IS (Aβ1–40 IS and Aβ1–42 IS) (Fig. 5A). Both the PAβ V1.0 and the PAβ V2.0 assay formats exhibited robust linearity across the tested concentration range, with r^2^ values for the linear regression lines exceeding 0.99 for both Aβ1–40 and Aβ1–42.

The inter-assay CV for both Aβ1–40 and Aβ1–42 were below 20% in the sample with the lowest non-zero concentrations. Thus, we set the LLOQs for both assays at 12.5 pg/ml for Aβ1–40 and at 3.125 pg/ml for Aβ1–42. Additionally, since the linearity extended to the sample with the highest concentrations, we set the upper limits of quantification (ULOQs) for the assays at 400 pg/ml for Aβ1–40 and 100 pg/ml for Aβ1–42.

#### Matrix effect assessment

To assess plasma matrix effect, we compared signals of Aβ peptides in plasma samples relative to BSA/PBS at three concentration levels (118.2pg/ml, 53.6pg/ml, and 21.4pg/ml for Aβ1–40, 47.2pg/ml, 23.0pg/ml and 10.8pg/ml for Aβ1–42) and calculated the matrix effect recovery following the formula outlined in the “Materials and Methods” section. Both the PAβ V1.0 and PAβ V2.0 assay formats demonstrated similar matrix effects (Table. 2, Fig. 4B). The detailed results are listed in Table 2.

Interestingly, we observed overall better recovery when using analyte specific IS to normalize peak area (Table. 2, Fig. 5B). These results suggest that different Aβ peptides might exhibit varying matrix effects, and analyte specific IS might be more robust in normalizing the matrix effect of corresponding analytes.

#### Assay precision

Assay precision was evaluated at three concentration levels (37.5 pg/ml, 146.4 pg/ml and 382.5 pg/ml for Aβ1–40, 82.8 pg/ml, pg/ml and 13.9 pg/ml for Aβ1–42) using normalized peak areas for both intra- and inter-assay assessments. The detailed results are listed in Table.3.

Similar %CVs were observed across both assays and normalization techniques, indicating strong reproducibility (%CV < 10%) for both PAβ V1.0 and PAβ V2.0 assays.

### 3.3 Relationship between plasma dilution and normalized intensity

The relationship between plasma dilution and normalized intensity for both the PAβ V1.0 and PAβ V2.0 assays was linear (r^2^ > 0.99), except for the Aβ1–42 of the PAβ V1.0 assay for which r^2^ was 0.758. This deviation can be attributed to the inaccuracy introduced by low S/N ratio at low concentration level (Fig. 4D).

### 3.4 Plasma volume requirement

The PAβ V1.0 assay was designed around the use of 250 μl plasma sample for each measurement. To test whether the PAβ V2.0 assay could enable measurement of Aβ peptides at lower plasma volumes, we examined both assays using varying amounts of plasma, ranging from 50 μl to 250 μl in increments of 50 μl, with three replicates for each sample volume. The results showed the PAβ V2.0 assays provided 178.0–22.7% higher S/N of Aβ1–40 and 87.6–26.1% higher S/N of Aβ1–42 from 50 μl to 250 μl (Fig. 4E).

Using a S/N ratio cutoff of 3, the PAβ V1.0 assay required a minimum of 100 μl to achieve quality measurement of Aβ1–40 and Aβ1–42, respectively, compared with 50–100 μl for the PAβ V2.0 assay (Fig. 4E).

### 3.5 IP recovery

To evaluate the proportion of Aβ peptides that were retained after the IP procedures, we utilized Simoa immunoassay to provide absolute quantification of Aβ peptides before and after IP IP recovery was evaluated at three concentration levels of low, medium, and high (27.4 pg/ml, 51.4 pg/ml, and 99.2 pg/ml for Aβ1–40; 7.0 pg/ml, 13.2 pg/ml, and 27.4 pg/ml for Aβ1–42) in triplicates. The result demonstrated that the PAβ V2.0 assay retained a higher proportion of Aβ peptides after IP (Fig. 4C).

### 3.6 Clinical assessment

#### Participant characteristics

In the PITT-ADRC cohort, the mean age was 75.6 years (SD 7.8), with 16 (53.3%) females. Nine participants (30.0%) carried the *APOE* ε4 genotype, and eight (26.7%) were diagnosed with probable AD. In terms of cognitive performance, the mean MMSE and MocA scores were 24.7 (SD 6.3) and 22.9 (SD 7.3), respectively. Regarding CDR scores, nine participants (30.0%) had a score of “disease absent” (CDR = 0), sixteen participants (53.3%) had a score of “questionable” (CDR = 0.5), three participants (10.0%) had a score of “disease present but mild” (CDR = 1), and two participants (6.7%) were categorized as “moderate” (CDR = 2). Comparing these metrics between the probable AD and normal control groups indicated significant differences in MoCA, MMSE and CDR scores. (Table 1)

In the AGUEDA cohort, the mean age was 71.4 years (SD 3.9), with 44 (57.1%) females. Twelve participants (16.0%) were *APOE* ε4 genotype carriers, eighteen (23.4%) were Aβ-PET positive and the averaged CL level was 7.5 (SD 25.2). The mean MMSE was 28.9 (SD 1.1), and MoCA score was 25.8 (SD 2.2). The average years of education was 11.7 (SD 4.8). Comparing these metrics between the Aβ-PET-positive and Aβ-PET-negative groups revealed no significant differences. The AGUEDA Aβ-PET data was also classified into three categories according to the CL scales: CL < 12 (Aβ-PET negative), 12 < CL < 24 (transition zone), and CL > 24 (Aβ-PET positive) [36, 37]. Comparing the metrics among the categories revealed significant differences in *APOE* ε4 genotype carriers. (Table. 1)

#### Clinical performance assessment

As mentioned in the Method section, we compared three different Aβ biomarkers: Aβ1–42/Aβ1–40 using the PAB V1.0 assay, Aβ1–42/Aβ1–40 using the PAβ V2.0 assay, and Aβ1–42/Aβ1–40 normalized with Aβ1–42 and Aβ1–40 IS correspondingly using the PAβ V2.0 assay.

**Figure 6:**
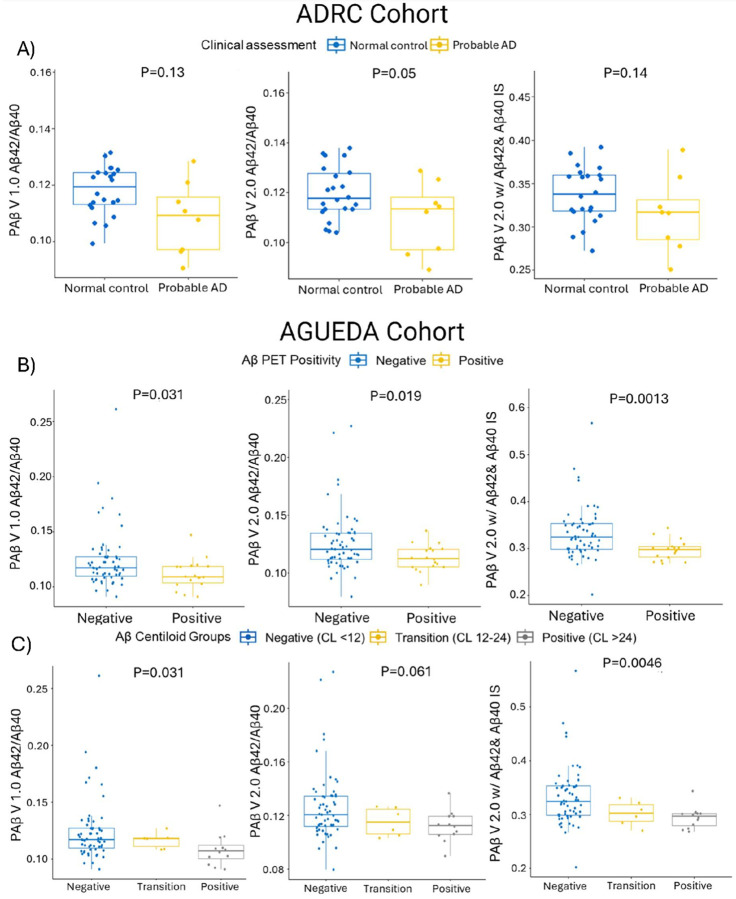
Clinical performance of plasma Aβ biomarkers. (A) Box and whisker plot categorizes the ADRC cohort into clinically assessed probable AD and normal control groups, analyzed using the Wilcoxon Rank Sum test, with p-values indicated. N represents the sample size. (B) Box and whisker plot shows the AGUEDA cohort split into Aβ PET positive and PET negative groups, analyzing three assay formats: PAβ V1.0 assay Aβ1–42/Aβ1–40, PAβ V2.0 assay Aβ1–42/Aβ1–40, and PAβ V2.0 assay Aβ1–42/Aβ1–40 normalized with Aβ1–40 IS and Aβ1–42 IS. Differences between groups were evaluated using the Wilcoxon Rank Sum test, with p-values provided. (C) Box and whisker plot dividing the AGUEDA cohort into CL positive, CL transition, and CL negative groups, with differences assessed using the Kruskal-Wallis test and p-values noted.

In the ADRC cohort, the PAβ V1.0, PAβ V2.0, and PAβ V2.0 with analyte-specific IS assays all showed equivalent performance in the Aβ1–42/Aβ1–40 ratio when comparing clinically assessed probable AD and normal control groups, with p-values of 0.13, 0.05, and 0.14, respectively (Fig. 6A). The effect sizes were consistent with those observed in the ADRC cohort, measuring 0.18, 0.20, and 0.30 for the PAβ V1.0, PAβ V2.0, and PAβ V2.0 with analyte specific IS assays, respectively.

In the AGUEDA cohort, significantly lower levels in the Aβ1–42/Aβ1–40 ratio were observed in Aβ-PET-positive versus Aβ-PET-negative groups using both PAβ V1.0 and PAβ V2.0 assays, with p-values of 0.031 and 0.019, respectively (Fig. 6B). Implementing analyte specific IS in the PAβ V2.0 assay revealed a similar performance, yielding a p-value of 0.0013. The effect sizes for the Aβ-PET-positive versus negative groups were 0.53, 0.56, and 0.73 for the PAβ V1.0, PAβ V2.0, and PAβ V2.0 with analyte specific IS assays, respectively.

The AGUEDA Aβ-PET data was also assessed according to the CL scales into three categories. In all assays, lower Aβ1–42/1–40 levels were observed in the Aβ-PET positive CL group, with p-values of 0.031 for PAβ V1.0, 0.061 for PAβ V2.0, and 0.0046 for PAβ V2.0 with analyte specific IS assays, respectively (Fig. 6C). The effect sizes for Aβ-PET-positive versus negative groups were 0.61, 0.59, and 0.76 for the PAβ V1.0, PAβ V2.0, and PAβ V2.0 with analyte specific IS assays, respectively.

These results indicate that the PAβ V2.0 assay with common IS normalization and analyte specific IS normalization performed comparably to the PAβ V1.0 assay in the clinical predictive performance.

### 3.7 Correlation between Aβ peptides measured in the PA p V1.0 and the PAβ V2.0 assays

To assess the measurement consistency between the PAβ V1.0 and PAβ V2.0 assays for Aβ peptides, we evaluated their correlation across two cohorts for Aβ1–42, Aβ1–40, Aβ1–39, Aβ3–40, Aβ1–38, and APP669–711. For both assays, Aβ1–39, Aβ3–40, Aβ1–38, and APP669–711 were normalized using the Aβ1–38 IS. Aβ1–42 and Aβ1–40 were normalized using both Aβ1–38 IS and analyte specific IS for the PAβ V2.0 assay, and Aβ1–38 IS only for the PAβ V1.0 assay. The correlation strength interpretation was based on previous publication [38].

**Figure 7:**
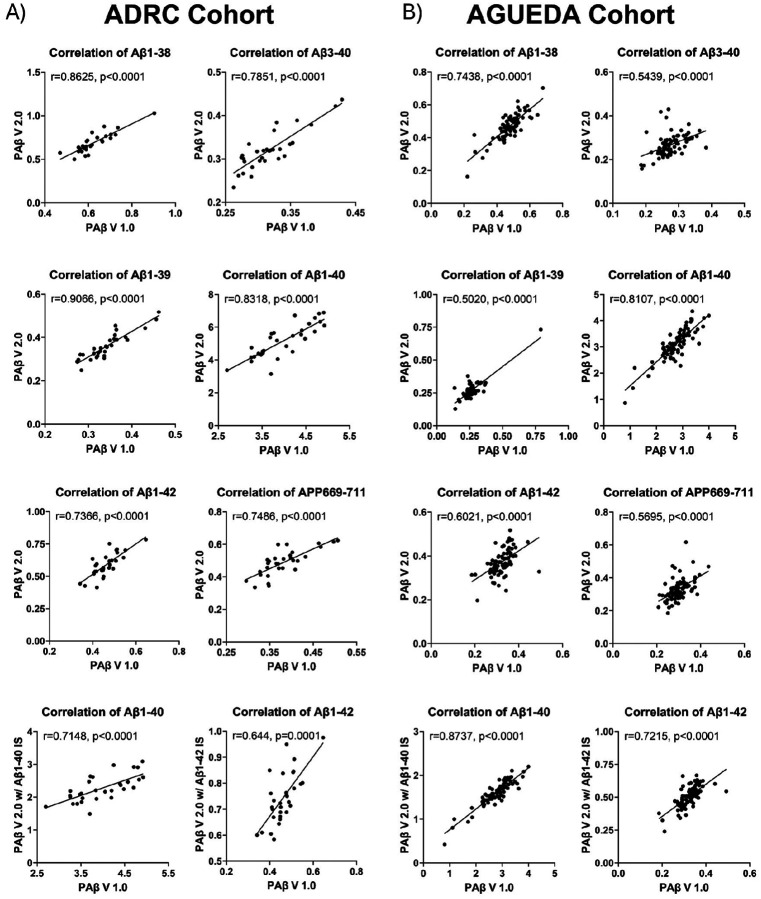
Correlation tests of PAβ V1.0 and PAβ V2.0 assays. The correlation between the PAβ V1.0 assay and PAβ V2.0 assay, normalized using Aβ1–38 IS, was illustrated for the ADRC (A) and AGUEDA (B) cohorts. Spearman correlation was employed to evaluate the strength of the correlation between Aβ peptide measurements across the two assays. Additionally, Aβ1–40 and Aβ1–42, normalized using Aβ1–40 IS and Aβ1–42 IS in the PAβ V2.0 assay, were further assessed for correlation with their respective Aβ peptides in the PAβ V1.0 assay, normalized using Aβ1–38 IS.

In the ADRC cohort, we observed strong correlations for Aβ1–38, Aβ1–39 and Aβ1–40 when normalized using Aβ1–38 IS (r > 8). The remaining Aβ peptides also exhibited strong correlations of 0.8 > r > 0.6 (Fig. 7A).

Similar results were obtained in the AGUEDA cohort, where there were strong correlations (r > 0.8) between Aβ1–40 measures when normalized using Aβ1–38 IS or Aβ1–40 IS. The correlation of Aβ1–42 (normalized using Aβ1–38 IS or Aβ1–42 IS) and Aβ1–38 was strong (0.8 > r > 0.6). Additionally, the correlation of the other Aβ peptides was moderate (0.6 > r > 0.5) (Fig. 7B), all indicating good agreement between the peptide levels measured in the different assay formats

## Discussion

Mounting evidence indicate that plasma Aβ ratio has utility to measure brain Aβ pathology [39], and target engagement in therapeutic programs targeting brain Aβ aggregates. It is critical for healthcare systems to utilize cost-effective and minimally invasive methods for clinical diagnosis and patient selection and monitoring for the recently approved immunotherapies. Additionally, Aβ is an early biomarker showing changes in patients with incipient disease including in cognitively normal older adults compared to non-diseased individuals, highlighting its critical role in the pre-clinical diagnosis of AD [40, 41]. Early detection provides an opportunity for intervention and potentially altering the disease course. Furthermore, implementing a blood-based biomarker test for patient triaging could potentially reduce the current 50-month wait for treatment access to just 12 months, as projected by specialist referral models for cognitive impairment and dementia patients [42]. These factors highlight the necessity of plasma-based IP-MS Aβ assay as a tool for early diagnosis.

Among the various blood-based Aβ assays, IP-MS methods such as the assay from Nakamura et al. stands out for its performance but has limitations needing improvement [22]. We adopted and enhanced this assay, resulting in the PAβ V2.0 assay with several enhancements. Firstly, our assay streamlined sample preparation time and preanalytical processing. Secondly, our new assay demonstrated a substantially stronger signal to noise ratio. Thirdly, the PAβ V2.0 and PAβ V1.0 assays exhibited similar clinical performance and analytical performance across multiple cohorts. To our knowledge, this is the first time that such significant enhancements have been achieved in refining the landmark Nakamura et al. plasma Aβ method.

The PAβ V2.0 assay successfully streamlined the IP steps using a commercially available buffer – the N4PE CSF diluent. The high detergent, high salt content and the interference blocker mixture in the buffer helped reduce the background noise. This buffer was selected after comparing its performance against several detergents and blocking buffers. While all other tested reagents exhibited lower S/N ratios compared with PAβ V1.0 assay, the N4PE diluent demonstrated higher S/N ratio, supporting its selection for further use as PAβ V2.0 assay.

The PAβ V2.0 assay maintained comparable analytical performance with a higher recovery rate compared to the PAβ V1.0 assay. This result was verified by SIMOA, an immunoassay with a different measurement mechanism than MS. We also tested the S/N of Aβ1–40 and Aβ1–42 in the PAβ V2.0 assay, utilizing a diluted sample volume of pooled plasma. The results demonstrated a higher S/N ratio and suggested the potential feasibility of decreasing the sample volume to 50–100 μl for the PAβ V2.0 assay. However, further investigation is warranted, including comparisons to Aβ PET imaging and/or CSF analysis, to assess the clinical utility and determine the feasibility of utilizing reduced sample volumes. Additionally, the PAβ V2.0 assay preserved similar clinical performance, with peptide concentrations showing strong correlation with those in the PAβ V1.0 assay.

We further tested the performance of using analyte specific IS (Aβ1–40 IS and Aβ1–42 IS) comparing to the common IS (Aβ1–38 IS) for normalization. Our results indicated that using analyte specific IS for normalization can slightly improve the matrix effect recovery of plasma Aβ peptides. However, the use of the analyte specific IS did not significantly change the analytical performance of the Aβ biomarkers. In the clinical performance analysis, regardless of whether the analyte specific IS was used in the PAβ V2.0 assay or not, the results did not show a significant improvement compared to the PAβ V1.0 assay. Our findings supported Nakamura et al.’s approach, confirming that using a common IS in the MALDI-TOF based IP-MS Aβ assay did not significantly alter clinical performance.

The MS instrument we utilized for our assays was a Bruker Microflex LT MALDI-TOF, widely adopted across numerous clinical facilities. Notably, it has received FDA approval for clinical microbiology diagnosis in humans [43, 44], and is widely available in many laboratories. In comparison to other MS instruments utilized in alternative IP-MS plasma Aβ assays, the Microflex is distinguished by its affordability and simplicity. Furthermore, it offers practical advantages, such as direct compatibility with a standard 110V outlet, without necessitating the use of any special electrical modifications or voltage converter. Moreover, its user-friendly interface facilitates straightforward operation, enabling general laboratory technicians to operate the instrument proficiently without requiring specialized training in mass spectrometry.

Our study has several notable strengths. Firstly, we describe in detail the technical development, analytical and clinical validation of an improved plasma Aβ assay by IP-MS. Secondly, our study included two different cohorts. This diverse representation enhances the generalizability and practical relevance of our findings. Thirdly, the cohorts had been characterized for biological evidence of disease using brain Aβ PET and neuropsychologically using established evaluation instruments such as the MMSE, MoCA, and CDR. Limitation of our study include the fact that the sample size across cohorts was relatively small. Future evaluations utilizing larger-scale studies, ideally conducted in real-world settings, will be necessary to validate and confirm the robustness of our findings.

In conclusion, we report successful development of a more resource-efficient and cost-effective IP-MS plasma Aβ assay. Compared with the in-house reproduced Nakamura et al. assay, the new assay demonstrated comparable clinical and analytical performance. The cost, time, and reagent savings, coupled with the utilization of a more affordable and widely available instrument, will enable research laboratories to conduct IP-MS analysis of Aβ in blood more effectively.

## Figures and Tables

**Table 1:** Demographic of participants

ADRC	Overall	Negative Control^§^	Probable AD^§^	p-value*				
Count	30	22	8					
Sex (% Female)	16 (53.3)	12 (54.5)	4 (50.0)	1.000				
Age (SD), year	75.6 (7.8)	75.3 (8.1)	76.6 (5.2)	0.530				
APOE ε4 carrier (%)	9 (30.0)	5 (22.7)	4 (50.0)	0.200				
MoCA score (mean (SD))	22.9 (7.3)	26.5 (2.2)	11.9 (6.7)	**<0.001**				
MMSE score (mean (SD))	24.7 (6.3)	27.6 (2.5)	16.9 (7.1)	**<0.001**				
CDR = 0 (%)	9 (30.0)	9 (40.9)	0 (0.0)	**<0.001**				
CDR = 0.5 (%)	16 (53.3)	11 (50.0)	5 (62.5)					
CDR = 1 (%)	3 (10.0)	2 (9.1)	1 (12.5)					
CDR = 2 (%)	2 (6.7)	0 (0.0)	2 (25.0)					
AGUEDA	Overall	Aβ-PET-negative^§^	Ap-PET-positive^§^	p-value*	<12 CL	12–24 CL	>24 CL	p-value*
Count	77	59	18		59	6	12	
Sex (% Female)	44 (57.1)	33 (55.9)	11 (61.1)	0.907	33 (55.9)	4 (66.7)	7 (58.3)	0.876
Age (SD), year	71.4 (3.9)	71.0 (4.0)	72.7 (3.3)	0.104	71.0 (4.0)	73.6 (3.6)	72.3 (3.1)	0.216
Education (mean (SD)), year	11.7 (4.8)	11.5 (5.0)	12.1 (4.2)	0.661	11.5 (5.0)	14.0 (4.1)	11.2 (4.1)	0.453
APOE ε4 carrier (%)	12 (16.0)	7 (12.1)	5 (29.4)	0.181	7 (12.1)	0 (0.0)	5 (41.7)	**0.024**
MOCA score (mean (SD))	25.8 (2.2)	25.8 (2.3)	25.9 (1.9)	0.737	25.8 (2.3)	26.5 (2.0)	25.7 (18)	0.709
MMSE score (mean (SD))	28.9 (11)	28.9 (1.0)	29.1 (1.4)	0.592	28.9 (1.0)	29.7 (0.5)	28.8 (1.7)	0.206
Centiloid (mean (SD))	7.5 (25.2)				−3.7 (8.25)	16.0 (3.0)	58.2 (22.3)	**<0.001**

Mean and Standard Deviation are reported for continuous variables. Frequencies and percentages are shown for categorical variables.

§ The diagnosis was performed by clinical diagnosis for ADRC cohort, and Aβ PET neuroimaging for AGUEDA cohort.

* P-values were calculated using the Wilcoxon Rank Sum or Kruskal-Wallis tests for continuous variables, and Fisher’s exact test for a categorical variable.

Two participants had missing data for APOE alleles. The percentage was calculated based on a sample size of 75.

Abbreviations: APOE, apolipoprotein E; CDR, Clinical Dementia Rating; MMSE, Mini Mental State Examination; MoCA, Montreal Cognitive Assessment; CL, Centiloid.

**Table 2:** Matrix effect recovery of the Aβ peptides using both normalization methods of Pittsburgh assays

PAβ V2.0 assay		Plasma, pg/mL	Matrix effect recovery with Aβ1–38 IS (%)	Matrix effect recovery with Aβ1–40 IS and Aβ1–42 IS (%)
Aβ1–40	Level 1	118.2	62.8	66.0
	Level 2	53.6	66.2	72.7
	Level 3	21.4	70.5	78.9
Aβ1–42	Level 1	47.2	57.8	63.4
	Level 2	23.0	62.3	76.9
	Level 3	10.8	69.8	85.4
PAβ V1.0 assay		Plasma, pg/mL	Matrix effect recovery with Aβ1–38 IS (%)	Matrix effect recovery with Aβ1–40 IS and Aβ1–42 IS (%)
Aβ1–40	Level 1	118.2	60.0	60.0
	Level 2	53.6	66.5	70.6
	Level 3	21.4	75.7	81.1
Aβ1–42	Level 1	47.2	55.8	62.0
	Level 2	23.0	65.2	78.9
	Level 3	10.8	74.5	89.1

**Table 3:** Inter- and Intra assay precision of the Aβ peptides using both normalization methods of Pittsburgh assays

		V2.0 assay (%)			
PAβ V2.0 assay		Intra-assay (n = 6)		Inter-assay (n = 5)	
Aβ1–40	pg/mL	Aβ1–38 IS Normalized	Aβ1–40 IS Normalized	Aβ1–38 IS Normalized	Aβ1–40 IS Normalized
Level 1	37.5	6.5	5.5	3.4	1.8
Level 2	146.4	7.9	7.0	5.9	2.6
Level 3	382.5	5.9	4.6	2.3	1.7
Aβ1–42	pg/mL	Aβ1–38 IS Normalized	Aβ1–42 IS	Aβ1–38 IS Normalized	Aβ1–42 IS Normalized
Level 1	82.8	6.8	4.6	4.3	4.8
Level 2	49.5	5.6	4.9	5.8	4.2
Level 3	13.9	4.1	5.3	4.2	3.0
Aβ1–42/Aβ1–40		Aβ1–38 IS Normalized	Aβ1–40 IS and Aβ1–42 IS Normalized	Aβ1–38 IS Normalized	Aβ1–40 IS and Aβ1–42 IS Normalized
Level 1	2.208	2.2	2.3	3.2	3.9
Level 2	0.338	5.2	5.1	6.6	6.6
Level 3	0.036	4.5	4.4	5.9	4.9
		V1.0 assay (%)			
PAβ V1.0 assay		Intra-assay (n = 6)		Inter-assay (n = 5)	
Aβ1–40	pg/mL	Aβ1–38 IS Normalized	Aβ1–40 IS Normalized	Aβ1–38 IS Normalized	Aβ1–40 IS Normalized
Level 1	37.5	3.4	1.9	4.9	0.8
Level 2	146.4	3.7	3.4	4.7	2.7
Level 3	382.5	6.7	4.4	2.6	3.0
Aβ1–42	pg/mL	Aβ1–38 IS Normalized	Aβ1–42 IS Normalized	Aβ1–38 IS Normalized	Aβ1–42 IS Normalized
Level 1	82.8	3.2	2.2	4.3	0.7
Level 2	49.5	2.8	3.4	5.3	2.6
Level 3	13.9	7.3	5.9	5.5	4.8
Aβ1–42/Aβ1–40		Aβ1–38 IS Normalized	Aβ1–40 IS and Aβ1–42 IS Normalized	Aβ1–38 IS Normalized	Aβ1–40 IS and Aβ1–42 IS Normalized
Level 1	2.208	1.5	2.6	2.0	2.6
Level 2	0.338	2.3	2.0	2.4	3.2
Level 3	0.036	4.2	4.1	5.3	4.6
